# Profiling Environmental Chemicals for Activity in the Antioxidant Response Element Signaling Pathway Using a High Throughput Screening Approach

**DOI:** 10.1289/ehp.1104709

**Published:** 2012-05-02

**Authors:** Sunita J Shukla, Ruili Huang, Steven O Simmons, Raymond R Tice, Kristine L Witt, Danielle VanLeer, Ram Ramabhadran, Christopher P Austin, Menghang Xia

**Affiliations:** 1 NIH Chemical Genomics Center, National Institutes of Health, Department of Health and Human Services, Rockville, Maryland, USA; 2 U.S. Environmental Protection Agency, Research Triangle Park, North Carolina, USA; 3 Division of the National Toxicology Program, National Institute of Environmental Health Sciences, National Institutes of Health, Department of Health and Human Services, Research Triangle Park, North Carolina, USA

**Keywords:** ARE, Nrf2, oxidative stress, qHTS, toxicity, Tox21

## Abstract

Background: Oxidative stress has been implicated in the pathogenesis of a variety of diseases ranging from cancer to neurodegeneration, highlighting the need to identify chemicals that can induce this effect. The antioxidant response element (ARE) signaling pathway plays an important role in the amelioration of oxidative stress. Thus, assays that detect the up-regulation of this pathway could be useful for identifying chemicals that induce oxidative stress.

Objectives: We used cell-based reporter methods and informatics tools to efficiently screen a large collection of environmental chemicals and identify compounds that induce oxidative stress.

Methods: We utilized two cell-based ARE assay reporters, β-lactamase and luciferase, to screen a U.S. National Toxicology Program 1,408-compound library (NTP 1408, which contains 1,340 unique compounds) for their ability to induce oxidative stress in HepG2 cells using quantitative high throughput screening (qHTS).

Results: Roughly 3% (34 of 1,340) of the unique compounds demonstrated activity across both cell-based assays. Based on biological activity and structure–activity relationship profiles, we selected 50 compounds for retesting in the two ARE assays and in an additional follow-up assay that employed a mutated ARE linked to β-lactamase. Using this strategy, we identified 30 compounds that demonstrated activity in the ARE-*bla* and ARE-*luc* assays and were able to determine structural features conferring compound activity across assays.

Conclusions: Our results support the robustness of using two different cell-based approaches for identifying compounds that induce ARE signaling. Together, these methods are useful for prioritizing chemicals for further in-depth mechanism-based toxicity testing.

Exposure to environmental stressors can induce oxidative stress in cells and result in a decrease in reducing potential and metabolic transformation to reactive intermediates (Nguyen et al. 2009; Simmons et al. 2011). Exogenous sources of oxidative stress include ionizing radiation, chemicals, and ultraviolet light, and endogenous sources include cellular signaling and metabolic processes or inflammation (Altieri et al. 2008; De Bont and van Larebeke 2004). Reactive oxygen species (ROS) induce damage to proteins, nucleic acids, and lipids leading to various cellular dysfunctions including apoptosis and necrosis (Simmons et al. 2011). Oxidative stress has been implicated in the pathogenesis of a variety of diseases ranging from cancer to neurodegeneration (Kobayashi 2010). In order to reduce the effects of oxidative stress, cells have developed adaptive stress response pathways involving induction of cytoprotective genes and repair of oxidant damage (Simmons et al. 2011).

The expression of many antioxidative enzymes is induced at the transcriptional level during oxidative stress and mediated by a *cis*-acting element, the antioxidant response element (ARE) (Friling et al. 1990). Overall, genes that are modulated by the ARE are involved in various aspects of cytoprotection, including producing antioxidants, inactivating ROS, and detoxifying xenobiotics (phase II enzymes). Despite the ability of several nuclear transcription factors such as Jun proteins (Dhakshinamoorthy and Jaiswal 2000; Jaiswal 1994; Venugopal and Jaiswal 1998) to bind ARE, activation of an ARE-mediated transcriptional response of downstream target genes is primarily mediated by nuclear factor E2-related factor 2 (Nrf2) (Jaiswal 2004; Moi et al. 1994).

Nrf2 is expressed in many tissues, including the liver, kidney, skin, lung, and gastrointestinal tract (Moi et al. 1994; Motohashi et al. 2002). Under normal cellular conditions, Nrf2 is sequestered in the cytoplasm by its negative regulator kelch-like ECH-associated protein 1 (Keap1) and maintained at low levels through ubiquitination and proteasomal degradation (Hur and Gray 2011). Keap1 cysteine residues act as ROS sensors and undergo a conformational change during oxidative stress conditions. This leads to the release and nuclear translocation of Nrf2, which then directs transcription of ARE-containing cytoprotective genes (Hur and Gray 2011). Because of its importance in disease prevention, the Nrf2 pathway is an attractive therapeutic target for high throughput screening efforts (Hu et al. 2010; Hur et al. 2010; Saw and Kong 2011; Tufekci et al. 2011).

Although the ARE pathway is important for compound detoxification, wide-scale testing of chemicals for their ability to induce this pathway has not occurred. One of the primary goals of the U.S. Tox21 initiative (Shukla et al. 2010) is the identification and prioritization of chemicals for further toxicological evaluation and development of predictive toxicity models of the *in vivo* response (Collins et al. 2008). During Tox21 Phase I, the National Institutes of Health (NIH) Chemical Genomics Center (NCGC) screened two compound libraries (each with approximately 1,400 compounds) provided by the U.S. National Toxicology Program (NTP) and the U.S. Environmental Protection Agency (EPA) in quantitative high throughput screening (qHTS) assays (Xia et al. 2008, 2009, 2011). The data generated have been used to identify the most robust assays for Tox21 Phase II, in which a library of > 10,000 compounds will be screened—initially across a battery of nuclear receptor and stress response pathway assays. Here, we report on a set of studies performed to assess the potential for compounds in the NTP Phase I library to induce the ARE pathway. We screened 1,408 compounds using two reporter gene-based assays in HepG2 cells. One assay utilized a β-lactamase reporter gene (the ARE-*bla* assay) and the other a luciferase reporter gene (the ARE-*luc* assay); the two assays differed in their ability to identify compounds that activate ARE through Nrf2-specific or nonspecific mechanisms. Selected compounds were retested in follow-up studies that included a mutated ARE reporter gene assay (where true active compounds should be inactive in this assay). Using this approach, we identified several known and novel inducers of ARE in addition to highlighting structural features of these compounds that confer activity across the assays.

## Materials and Methods

**Figure 1:**
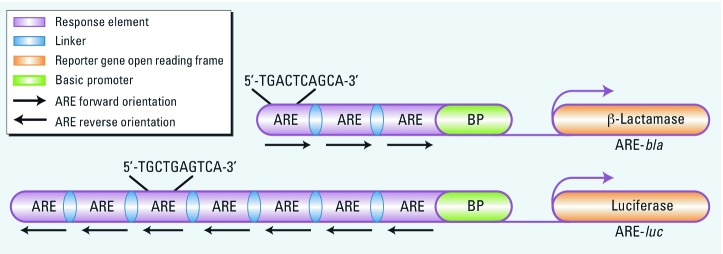
Schematics of ARE-*bla* and ARE-*luc* reporter gene assays. The ARE-*bla* reporter harbors three AREs derived from the human *NQO1* gene upstream of a basic (minimal) promoter that drives the expression of b-lactamase. The ARE-*luc* reporter gene harbors seven multimerized inverted consensus AREs upstream of a synthetic basic (minimal) promoter, which contains only Nrf2 binding sequences and CCAAT and TATA boxes that drive the expression of firefly luciferase.

*Cell culture and conditions.* The Invitrogen CellSensor® ARE-*bla* HepG2 cell line (Life Technologies, Madison, WI), contains three stably integrated copies of the ARE derived from the reduced form of human nicotinamide adenine dinucleotide phosphate (NADPH) quinone oxidoreductase 1 gene (*NQO1*) (Dhakshinamoorthy and Jaiswal 2000) driving the expression of a downstream β-lactamase reporter gene. The ARE-*luc* HepG2 cell line has been previously described (Simmons et al. 2011). Briefly, a Nrf2-responsive luciferase reporter gene was engineered to specifically measure Nrf2-dependent transcriptional activity. In an effort to identify artifacts associated with the ARE-*bla* assay, such as fluorescence (Simeonov et al. 2008), we used the ARE-*bla*-mut assay (Simmons et al. 2011) in follow-up studies. All assay conditions are further described in Supplemental Material, p. 2 (http://dx.doi.org/10.1289/ehp.1104709). The molecular characteristics of these three assays are provided in Figure 1 and Table 1.

**Table 1:** Cell-based assays used in the antioxidant response element (ARE) profiling and follow-up studies.

Assay characteristics	ARE-bla	ARE-luc	ARE-bla-mut
Reporter		b-Lactamasea		Luciferasea		b-Lactamase
Cell lineage		Monoclonal		Polyclonal		Polyclonal
No. of AREs		3		7		7
ARE type		Expandedb		Coreb		Mutant
ARE spacing		15 bp		12 bp		12 bp
ARE helical turn		180°		72°		72°
ARE orientation		Senseb		Antisenseb		NA
Basal promoter		Minimal viral		Synthetic		Synthetic
Abbreviations: bp, base pair; NA, not applicable. ab-Napthoflavone (46 µM–1.4 nM) used as positive control in primary screening assay. bDefined by Nerland et al. (2007).						

*ARE reporter gene assays.* ARE-*bla*, ARE-*luc*, and ARE-*bla*-mut HepG2 cells were resuspended in assay medium (growth medium plus 1% dialyzed fetal bovine serum) and dispensed at 2,000 cells/5 µL/well. Cells were plated in 1,536-well black wall/clear bottom plates (Greiner Bio-One North America, Monroe, NC) for ARE-*bla* and ARE-*bla*-mut assays and in 1,536-well white wall/solid bottom plates (Greiner) for the ARE-*luc* assay using a Flying Reagent Dispenser (Aurora Discovery, Carlsbad, CA). After incubation at 37°C for 6 hr to allow cell attachment to the well bottom, 23 nL of compound dissolved in dimethyl sulfoxide (DMSO) or DMSO only was added to the assay plates via pintool (Kalypsys, San Diego, CA); plates were then incubated for an additional 16 hr overnight (exposure duration was determined for optimal expression of β-lactamase after performing several time course experiments; data not shown). The next day (for the ARE-*bla* and ARE-*bla*-mut assay), 1 µL of LiveBLAzer™ (Invitrogen; Life Technologies) detection mix was added to each well and the plates were subsequently incubated at room temperature in the dark for 2 hr. Fluorescence intensity after 405 nm excitation was measured at 460 and 530 nm emissions by an Envision plate reader (PerkinElmer, Boston, MA). Data were represented as the ratio of the 460/530 emission values. To measure ARE induction using the luciferase reporter readout, 5 µL One-Glo luciferase reagent (Promega, Madison, WI) was added to each well. After a 30-min room temperature incubation, plates were read on a ViewLux plate reader (PerkinElmer) using a 20-sec exposure time.

*The NTP 1,408 compound library and compound profiling.* The NTP collection of 1,408 compounds has been previously described (Xia et al. 2008). Compound reproducibility within each assay was calculated using the 66 replicate compounds in the NTP library (Huang et al. 2011), leaving 1,340 unique compounds. All compounds were prepared as 10-mM stock solutions and screened at 14 concentrations. Final compound concentrations ranged from 0.59 nM to 92 µM. To achieve the 92-µM concentration, 23 nL of compound was transferred twice from the highest concentration compound plate into each well of the assay plates. A total of 18 plates, including DMSO-only plates, were tested for the ARE-*bla* and ARE-*luc* assays. β-napthoflavone, a known ARE inducer (Dhakshinamoorthy and Jaiswal 2000; Dewa et al. 2008), was used as a positive control. The control well layout for the primary screening is described in Supplemental Material, pp. 2–3 (http://dx.doi.org/10.1289/ehp.1104709).

*Data analysis and curve fitting.* Data analysis and curve fitting were performed as previously described (Xia et al. 2008). Raw plate reads for each titration point were normalized to the maximal β-napthoflavone control response (100%) and DMSO-only wells (0%, basal). Because of the unavailability of good positive controls for the ARE-*bla*-mut follow-up assay, all data were normalized to DMSO wells (0%, basal) and ARE-*bla* wells (100%, maximal β-napthoflavone control response). Concentration–response data for each compound were fitted to a four-parameter Hill equation,


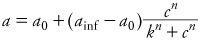


where *a* is compound activity (percent), *c* is compound concentration, *a*_0_ is compound activity at zero concentration, *a*_inf_ is compound activity at infinite concentration, *k* is half-maximal activity (EC_50_), and *n* is the Hill coefficient. *a*_0_, *a*_inf_, *k*, and *n* are the four parameters derived from the curve–fitting process. This process yielded concentrations of EC_50_ and maximal response (efficacy) values for each concentration response curve (CRC).

CRCs were classified into four major groups as previously described (Huang et al. 2011). Curve class 1 compounds show complete response, whereas curve class 2 compounds show partial response. Class 1 and 2 curves are further subdivided into subclasses based on efficacy and quality of fit (*R*^2^), where curve class 1.1 and 2.1 curves show > 80% efficacy and an *R*^2^ of > 0.9. Curve classes 1.2 and 2.2 show 30–80% efficacy and an *R*^2^ of > 0.9. Thus, curve classes 1.1, 1.2, 2.1, and 2.2 are the highest confidence curves associated with compound activity. Other curves and subclasses show either single-point activity (class 3) or have lower efficacies and *R*^2^ values (30–80% and < 0.9, respectively) and hence are associated with inconclusive activity. Class 4 curves do not exhibit a CRC and are inactive.

Hierarchical clustering of compound activity patterns was performed with Spotfire DecisionSite version 8.2 (TIBCO Spotfire Inc., Cambridge, MA) using correlation of the log EC_50_ values as the similarity metric across the three follow-up assays [see Supplemental Material, Figure S1 (http://dx.doi.org/10.1289/ehp.1104709)].

*Structure–activity relationship (SAR) analysis and follow-up studies.* The NTP 1408 compound structures were first converted into 2,048-bit Daylight® fingerprints (Daylight Chemical Information Systems Inc., Laguna Niquel, CA) and then clustered using the Self-Organizing Map algorithm (Kohonen 2006), resulting in 285 clusters. A total of 63 compounds were selected for confirmation and follow-up studies from the clusters that contained compounds that were active in the ARE-*bla* or ARE-*luc* assays. The detailed selection process was as follows. There were 8 clusters each containing at least 2 compounds that were active in both the ARE-*bla* and the ARE-*luc* assays (these were defined as common actives). These common actives were selected. In addition, 1–2 compounds were selected from each of the 8 clusters that were structurally closely related to the common actives but were either active only in the ARE-*bla* assay or inactive in both assays. There were 16 clusters that each contained only 1 common active, which were selected. Of the clusters that contained compounds that were active only in the ARE-*bla* assay, 6 contained at least 3 ARE-*bla* only actives. From each of these clusters, 1–2 actives were selected along with 1 inactive that was structurally closely related to the actives. Finally, 2 compounds that were active only in the ARE-*luc* assays were also selected.

Follow-up studies were performed to confirm sample integrity and assay reproducibility. Of the 63 compounds that passed quality control measures [see Supplemental Material, Figure S2 (http://dx.doi.org/10.1289/ehp.1104709)], 50 were cherry-picked from the original solutions provided by NTP. The compounds were prepared in duplicate 12-point, 3-fold dilution titrations in DMSO, with final concentrations ranging from 0.26 pM to 46 µM. Compounds that were curve class 1.1, 1.2, 2.1, or 2.2 (i.e., active) in the primary assay and curve class 4 (i.e., inactive) in the follow-up assay (or vice versa) were not considered confirmed; all other compounds were considered confirmed.

## Results

*Identification of environmental compounds that induce the ARE pathway.* Both the ARE-*bla* and ARE-*luc* cell-based assays performed well, with average Z´ factors (Zhang et al. 1999) of 0.71 and 0.69, respectively. β-Napthoflavone, a known ARE inducer, replicated well across all 18 plates for both assays with average EC_50_ values of 2.1 µM and 5.2 µM in the ARE-*bla* and ARE-*luc* assays, respectively. Because there were 66 duplicate compounds represented in the NTP collection, we calculated reproducibility for both ARE-*bla* and ARE-*luc* assays using the ratio readout. There was a 97% and 100% concordance rate for the 66 duplicate compounds for the ARE-*bla* and ARE-*luc* assays, respectively. In the ARE-*bla* assay, the EC_50_ and efficacy values of the high-quality replicates (*n* = 17) correlated well with *R*-values of 0.94 and 0.70, respectively. The EC_50_ and efficacy values for the high-quality (curve classes 1.1, 1.2, 2.1, 2.2) replicates (*n* = 3) in the ARE-*luc* assay were well correlated, with *R*-values of 0.99 and 0.95, respectively.

Of the 1,340 unique NTP compounds, 388 (29%) high-quality ARE pathway inducers were identified in the ARE-*bla* assay and 44 (3%) were identified as Nrf2-specific ARE pathway inducers in the ARE-*luc* assay [see Supplemental Material, Tables S1,S2 (http://dx.doi.org/10.1289/ehp.1104709)]. There were 34 high-quality active compounds that were common between the ARE-*bla* and ARE-*luc* assays (see Supplemental Material, Table S3). The majority of the 34 compounds had average potency values of 25 µM (ARE-*bla*) and 37 µM (ARE-*luc*) and included known ARE inducers such as curcumin (Balstad et al. 2011) and acetochlor (Rakitsky et al. 2000).

*Confirmation of ARE pathway inducers.* To confirm the activity of a subset of compounds based on activity profiles from the primary screening, the 50 compounds that passed quality control measures were retested in the original ARE assays and the ARE-*bla*-mut assay to identify promiscuous β-lactamase activators. Of the 50 compounds retested in the ARE-*bla* and ARE-*luc* assays, 47 (94%) and 45 (90%), respectively, had confirmed activity between the primary screening and follow-up studies. Three nonconfirming compounds (melphalan, 4-chloro-*o*-phenylenediamine, and 1,10-phenanthroline monohydrate) all showed activity in the primary ARE-*bla* assay but were deemed inactive upon follow-up screening. Of the 5 nonconfirming compounds, 3 (rhothane, *o*-aminophenol, and lithocholic acid) were inactive in the primary ARE-*luc* assay and active in the follow-up assay, and 2 (*N*-isopropyl-*n*´-phenyl*-p*-phenylenediamine and *n*-(1,3-dimethylbutyl)-*n*´-phenyl-*p*-phenylenediamine) were active in the primary ARE-*lu*c assay and inactive in the follow-up assay.

The activity profiles of the compounds across each follow-up assay are shown in the heat map in Supplemental Material, Figure S1 (http://dx.doi.org/10.1289/ehp.1104709), where 36/50 (72%) and 32/50 (64%) were active in the ARE-*bla* and ARE-*luc* assays, respectively. Of the 50 compounds that were retested, 30 (60%) were active across both assays (see Supplemental Material, Table S4) and inactive in the ARE-*bla*-mut assay except for 4 compounds that may exhibit autofluorescence (benzo[*b*]fluoranthene, benzo[*k*]fluoranthene, D&C Yellow II, and iodochlorohydroxyquinoline).

**Figure 2:**
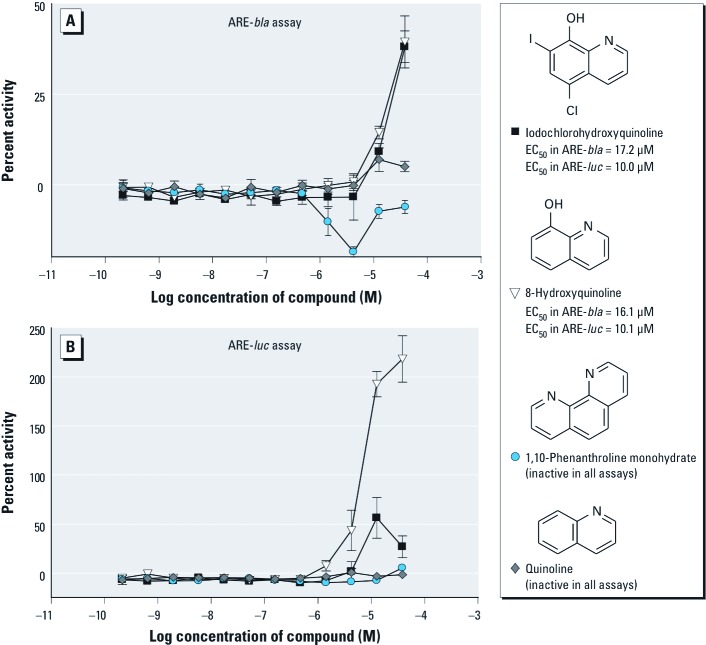
Compounds from cluster 1 with selective activity in the ARE-*bla* (*A*) and ARE-*luc* (*B*) assays. Compounds shown were chosen for follow-up studies and tested across the two ARE reporter gene assays. Each concentration response curve and EC_50_ value represents the mean ± SD response of ARE-*bla* (*n* = 5) or ARE-*luc* (*n* = 3) determinations.

**Figure 3:**
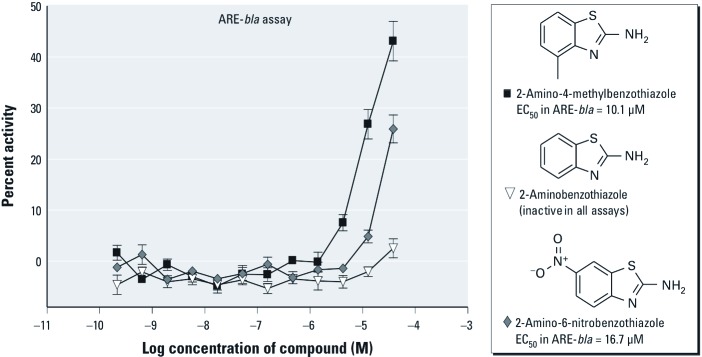
Compounds from cluster 5 with selective activity in the ARE-*bla* assay. Compounds shown were chosen for follow-up studies and tested across ARE reporter gene assays. Each concentration response curve and EC_50_ value represents the mean ± SD response of ARE-*bla* assay determinations (*n* = 5). Compounds were inactive in the ARE-*luc* assay (data not shown.)

**Figure 4:**
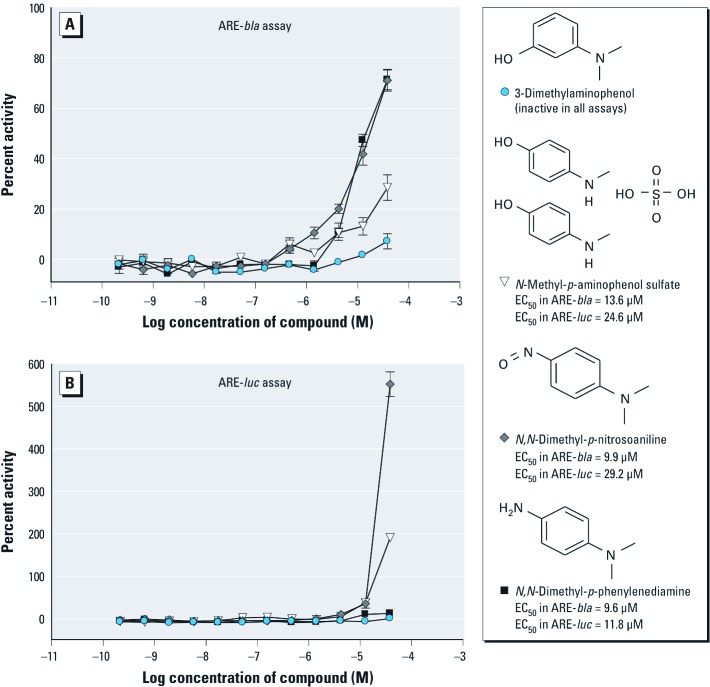
Compounds from cluster 7 with selective activity in the ARE-*bla* (*A*) and ARE-*luc* (*B*) assays. Compounds shown were chosen for follow-up studies and tested across ARE reporter gene assays. Each concentration response curve and EC_50_ value represents the mean ± SD response of ARE-*bla* (*n* = 5) or ARE-*luc* (*n* = 3) determinations.

**Figure 5:**
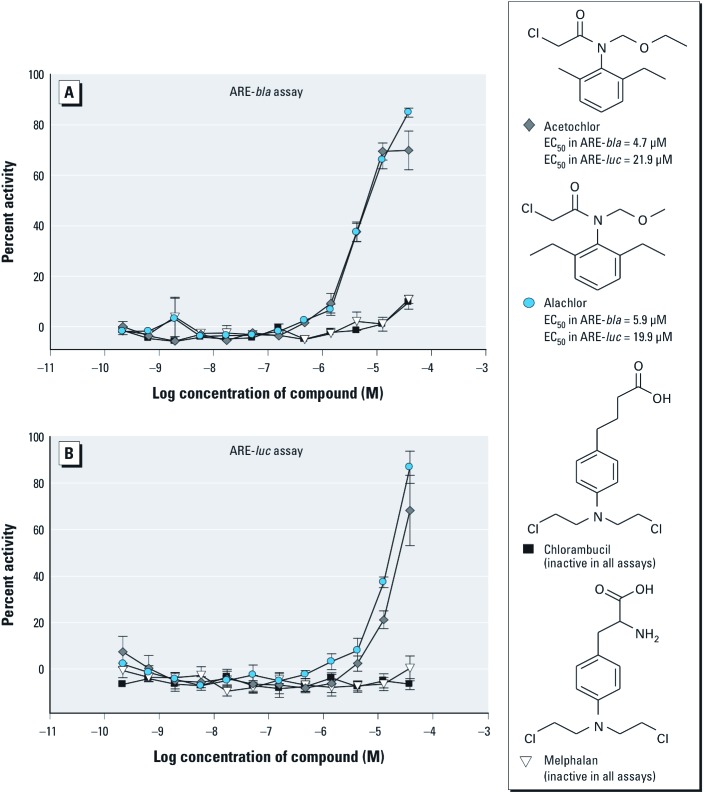
Compounds from cluster 8 with selective activity in the ARE-*bla* (*A*) and ARE-*luc* (*B*) assays. Compounds shown were chosen for follow-up studies and tested across ARE reporter gene assays. Each concentration response curve and EC_50_ value represents the mean ± SD response of ARE-*bla* (*n* = 5) or ARE-*luc* (*n* = 3) determinations.

*SAR of ARE pathway inducers.* Fifty compounds representing 26 clusters (with ≥ 1 compounds per cluster) [see Supplemental Material, Table S4 (http://dx.doi.org/10.1289/ehp.1104709)] were chosen for follow-up on the basis of structural similarity and activity patterns across the ARE-*bla* and ARE-*luc* assays. Further interrogation of each cluster revealed various activity patterns within each cluster (i.e., active in the ARE-*bla* assay only, active in the ARE-*luc* assay only, or active in both assays) (see Supplemental Material, Table S4). Analysis of activity patterns combined with chemical structure information may provide insight into why certain compounds within the same cluster show differential activity among the ARE assays. Cluster 1 contains iodochlorohydroxyquinoline, 8-hydroxyquinoline, 1,10-phenanthroline monohydrate, and quinoline (Figure 2). Iodochlorohydroxyquinoline and 8-hydroxyquinoline were active in both ARE assays, with iodochlorohydroxyquinoline being the only compound from this cluster active in the ARE-*bla*-mut assay [see Supplemental Material, Table S4 (http://dx.doi.org/10.1289/ehp.1104709)]. However, the efficacy of iodochlorohydroxyquinoline in the ARE-*bla*-mut assay was lower (21%) than in the ARE-*bla* assay (41%), suggesting ARE-*bla* activity. Both quinoline and 1,10-phenanthroline monohydrate were inactive across all assays. Cluster 5 contains three compounds (Figure 3, and see Supplemental Material Table S4): 2-amino-4-methylbenzothiazole and 2-amino-6-nitrobenzothiazole were active only in the ARE-*bla* assay, whereas 2-aminobenzothiazole was inactive in all assays. Cluster 7 contains 3-dimethylaminophenol, *N*-methyl-*p*-aminophenol sulfate, *N*,*N*-dimethyl-*p*-nitrosoaniline, and *N*,*N*-dimethyl-*p*-phenylenediamine (Figure 4). Only 3-dimethylaminophenol was inactive in both the ARE-*bla* and ARE-*luc* assays. The 1,4-substitutions, as opposed to a 1,3-substitution, on the phenyl ring could be important for these compounds’ ARE activity. Cluster 8 contains acetochlor, alachlor, chlorambucil, and melphalan (Figure 5). Whereas acetochlor and alachlor were active in both the ARE-*bla* and ARE-*luc* assays, chlorambucil and melphalan were inactive across all assays. Although sharing a common phenylamine substructure, these four compounds can be further divided into two subgroups—with acetochlor and alachlor in one group sharing the larger 2-chloro-*N*-(2,6-dimethylphenyl)-*N*-(methoxymethyl)-acetamide core, and chlorambucil and melphalan in the other group, sharing the larger *N*,*N*-bis(2-chloroethyl)aniline core. The acetamide-containing group may be important for inducing ARE activity.

## Discussion

The strategy reported here is a comprehensive utilization of qHTS, multiple cell-based approaches, SAR, and cluster analysis as a first step in the identification of chemicals that induce the ARE signaling pathway. Specifically, we used two different reporter-based assays to profile 1,408 compounds for their ability to induce the ARE pathway [the entire strategy is summarized in Supplemental Material, Figure S2 (http://dx.doi.org/10.1289/ehp.1104709)]. The ARE-*bla* reporter was constructed using an enhancer harboring three serial repeats of the human *NQO1* ARE sequence capable of binding various nuclear transcription factors (Dhakshinamoorthy and Jaiswal 2000), including Nrf2. Thus, this assay potentially detects ARE activators through Nrf2-dependent and independent mechanisms. The ARE-*luc* reporter was constructed using entirely synthetic sequences designed to identify ARE activators operating only through a Nrf2-dependent mechanism. Among the 1,340 unique compounds in the primary screen, we identified 388 (29%) and 44 (3%) actives in the ARE-*bla* and ARE-*luc* assays, respectively. Fifty compounds were chosen for follow-up studies on the basis of SAR and biological activity profiles, with 90–94% of these compounds confirming activity in follow-up screening. Out of the 50 compounds retested, 3 were active in primary screening and inactive upon follow-up testing, giving a 6% false positive rate in the ARE-*bla* assay. The false positive and false negative (compounds that were active in follow-up testing) rates for the ARE-*luc* assay were 4% (2/50) and 6% (3/50), respectively. As expected, the majority of compounds (78%, 34/44) that were active in the ARE-*luc* assay were also active in the ARE-*bla* assay upon follow-up testing. Thus, a number of chemicals were confirmed to operate through an ARE-dependent manner as evidenced through the use of two different reporter genes.

The utilization of different assay formats facilitated the identification of pharmacological characteristics associated with the ability or inability to activate Nrf2 transcriptional activity in one or both assays. Marked differences existed in the primary screening results between the number of active compounds identified in the ARE-*bla* and ARE-*luc* assays. As seen in Table 1, there are differences between the two cell-based assays employed, including the reporter, cell lineage (monoclonal vs. polyclonal), number of ARE sequences located in the enhancer regions and their orientation, and basal promoter. One main difference between the two cell-based models is that the ARE sequence for the ARE-*bla* line is derived from the *NQO1* gene, whereas the ARE sequence for the ARE-*luc* line is derived from the heme oxygenase (decycling) 1 gene (*HMOX1*). Both of these genes are well-characterized Nrf2 target genes: *NQO1* contains ARE sequences in the sense direction, and *HMOX1* contains ARE sequences in the antisense direction (Nerland 2007). ARE elements associated within the *HMOX1* promoter are located much farther upstream of the transcription start site (Knorr-Wittmann et al. 2005), whereas the ARE element found in *NQO1* is located more proximal to the start site (Nioi et al. 2003). The different ARE target genes used for the promoter and enhancer sequences resulted in either sense (*NQO1*, ARE-*bla*) or antisense (*HMOX1*, ARE-*luc*) orientation of the ARE response elements. Additionally, the proximity of the antiparallel ARE sequences to the reporter transcription start sites may affect transcription and sensitivity of a particular assay.

Another key difference between the assays is the monoclonal and polyclonal origins of the ARE-*bla* and ARE-*luc* cell lines, respectively. The integration site(s) of the reporter transgene has a stronger influence on reporter performance/fidelity in monoclonal lines because of the single integration pattern, which could enhance or pervert reporter gene function. In other words, the integration site for the selected monoclonal cell line may provide access for Nrf2, or it may harbor sites for other DNA-binding proteins (non-ARE specific) that affect reporter function. In terms of a virally derived (ARE-*bla*) or synthetically derived (ARE-*luc*) minimal promoter, viral promoters are bound by ubiquitous transcription factors such as NFκB and SP1 and can induce β-lactamase expression in a Nrf2-independent manner (Bakovic et al. 2000; Hiscott et al. 2001). Because the ARE-*luc* was designed to eliminate the predicted binding sites for nearly all non-Nrf2 DNA-binding proteins, it is expected that the ARE-*luc* assay would detect fewer ARE actives. It is also possible that Nrf2 interacts with other transcription factors bound to a virally derived minimal promoter to more effectively stimulate reporter expression in response to oxidative stress in comparison with a synthetic promoter. Overall, differences in sensitivity between the two assays resulting from promoter/enhancer construction, ARE orientation, and other factors may explain discrepancies in the results and predispose the ARE-*bla* assay to false positives and ARE-*luc* assay to false negatives. Thus, it may be beneficial to use both assays for identifying compounds that induce oxidative stress.

Even though a much larger library of 1.2 million small molecules has been screened in HTS for ARE inducers (Hur et al. 2010), this is the first study to profile a large set of mostly environmental compounds in a qHTS format (for primary and follow-up screening) to identify inducers of the ARE pathway and, by extrapolation, compounds capable of inducing oxidative stress. The use of a paired cell line approach in addition to an ARE-*bla* mutant assay helped to confirm compounds that operate through an ARE-Nrf2 based mechanism. Furthermore, SAR and cluster analysis identified known (e.g., quinoline compounds) and novel compounds, where the identification of known compounds confirms the use of this approach as a tool for prioritizing compounds in follow-up studies. This approach could be used also to evaluate the bioactivity profile of a large number of mixtures (Simmons et al. 2009; Tal et al 2010), and in the next phase of Tox21, a number of mixtures as well as each individual constituent will be screened as part of the Tox21 10,000-compound library in these assays. Since oxidative stress has been associated with a number of diseases, we attempted to identify structural features of compounds from different structure classes and activity groups to obtain a better understanding of the mechanism of ARE pathway activation.

While it is reasonable to assume that the compounds identified by this approach induce ARE activity through oxidative stress, it might be possible that Nrf2 can be induced through an unrelated mechanism. For example, resveratrol and l-sulforaphane, compounds active in the ARE assays used in this study, are generally considered to be antioxidants (Kelsey et al. 2010). However, consistent with the Nrf2 response, resveratrol has been shown to induce DNA damage *in vitro* over the same concentration range tested here (Fox et al. 2012).

We identified two benzothiazole compounds that only demonstrated activity in the ARE-*bla* assay (Figure 3). Benzothiazoles have various known biological properties including antimicrobial and anti-tumorigenic activity (Kamal et al. 2009; Leong et al. 2003; Yoshida et al. 2005). Our results indicate that phenyl ring substitutions on the 2-aminobenzothiazoles may be necessary to confer the type of activity observed in the ARE-*bla* assay. However, 2-amino-4-methylbenzothiazole and 2-amino-6-nitrobenzothiazole have not previously been reported as ARE inducers and may need further investigation to clarify their activity. There were three compounds in cluster 7 [Figure 4, Supplemental Material, Table S4 (http://dx.doi.org/10.1289/ehp.1104709)] that demonstrated activity in the ARE-*bla* and ARE-*luc* assays. *N*,*N*-dimethyl-*p*-phenylenediamine produces a long-lived radical cation, which may induce oxidative stress (Verde et al. 2002). *N*-methyl-*p*-aminophenol sulfate and *N*,*N*-dimethyl-*p*-nitrosoaniline may be novel compounds with regard to their role in oxidative stress. Cluster 8 contained two compounds, acetochlor and alachlor, which demonstrated activity in both ARE-*bla* and ARE-*luc* assays (Figure 5). Both compounds are commonly used herbicides, believed to share a common mechanism of toxicity based on their ability to cause nasal tumors (Wilson et al. 2011). Furthermore, genomic data obtained on the olfactory mucosa of rats treated with alachlor indicate up-regulation of genes associated with the generation of ROS and resulting oxidative damage (Genter et al. 2002). Cluster 1 also contained two compounds that demonstrated activity in all three assays (iodochlorohydroxyquinoline) or in both the ARE-*bla* and ARE-*luc* assays (8-hydroxyquinoline) (Figure 2, Supplemental Material Table S4). Although iodochlorohydroxyquinoline demonstrated activity in the ARE-*bla*-mut assay, the efficacy (21%) was considerably lower than in the ARE-*bla* assay (40%), supporting the activity of iodochlorohydroxyquinoline in the ARE pathway. The generation of ROS has been confirmed in cell-based testing (Chen et al. 2009), and this compound was noted to activate hypoxia-inducible factor 1 in a β-lactamase reporter gene assay (Xia et al. 2009), further supporting iodochlorohydroxyquinoline activity in the ARE pathway. 8-Hydroxyquinoline, used in the development of pesticides and herbicides (Li et al. 2010), has been shown to induce the mitogen-activated protein kinase (MAPK) pathway in HeLa cells, a pathway important in oxidative stress-mediated cell death (Chen et al. 2009). The halide substituents may be associated with the promiscuous behavior of iodochlorohydroxyquinoline in the β-lactamase assays that is due to activity in the ARE-*bla*-mut assay, whereas the hydroxyl group in 8-hydroxyquinoline appears to be responsible for its activity in the ARE-*bla* and ARE-*luc* assays (and for the activity of iodochlorohydroxyquinoline in the ARE-*luc* assay). In both of these cases, the hydroxyl group appears to be important for conferring activity.

## Conclusions

The ARE-*bla* assay may identify compounds that induce ARE through complex interactions of multiple transcription factors, whereas the ARE-*luc* assay may capture the activity of Nrf2 in response to oxidative stress. The use of multiple reporter-based assays to test ARE pathway activity has the potential to reveal compound-specific signatures that can be used to further cluster chemicals on the basis of biological activity profiling (Simmons et al. 2009). Indeed, we were able to identify structural features of several compounds within the same cluster that conferred differences in activity profiles; this information may potentially be used to predict the ability of other compounds to induce the ARE pathway.

Thus, primary screening assays such as those employed in this study can facilitate the simultaneous evaluation of thousands of chemicals over a broad concentration range. Detailed mechanistic information obtained from lower throughput, higher content assays are the next steps to elucidate mechanism of action. In such studies, the effect of compounds on the ARE pathway would be assessed in relevant cells/tissues exposed to environmentally relevant concentration ranges encompassing likely human exposures. Furthermore, it would be useful to determine whether compounds active for inducing ARE are active in other stress response pathway assays, such as those that measure DNA damage, inflammation, and ER stress. Additional studies may also include characterization of Nrf2 translocation for compounds that only showed activity in the ARE-*bla* assay in order to assess Nrf2 and non-Nrf2-mediated ARE induction. Additional downstream assays, such as measuring glutathione depletion, may provide support for the utility of the pathway-based screening approaches described here.

## Supplemental Material

(831 KB) PDF
