# Supplementary material for: Profiling Environmental Chemicals for Activity in the Antioxidant Response Element Signaling Pathway Using a High Throughput Screening Approach
Source: Environ Health Perspect. 2012 May 2;120(8):1150–6. doi: 10.1289/ehp.1104709 (PMC3440086; doi:10.1289/ehp.1104709)
Supplement: (831 KB) PDF [file ehp.1104709.s001.pdf]

Supplemental Material

Profiling Environmental Chemicals for Activity in the Antioxidant Response Element Signaling Pathway Using a High-Throughput Screening Approach

Sunita J. Shukla, Ruili Huang, Steven O. Simmons, Raymond R. Tice, Kristine L. Witt, Danielle VanLeer, Ram Ramabhadran, Christopher P. Austin, and Menghang Xia

Table of Contents

Cell Culture and Conditions 2
Control well layout for the primary screening 2

Chemical Analysis for Compound Purity 3
References 16

Tables
Table S1. Activities and potencies for compounds from primary qHTS screen 4

Table S2. Activities and potencies for compounds from primary qHTS screen 5

Table S3. Activities and potencies for 34 compounds overlapping ARE-*bla* and ARE-*luc* assays in the primary qHTS screen 6

Table S4. Potencies (µM) and efficacies (%) of compounds from ARE confirmation studies 7

Figures
Figure S1. Heat map of all follow-up compounds tested in ARE-*bla*, ARE-*bla*-mut, and ARE-*luc* assays. 14

Figure S2. A flowchart of identification of ARE inducers in multiple assay formats using qHTS 15

Cell Culture and Conditions

For the ARE-*bla*-mut assay, a stable ß-lactamase reporter cell line utilizing the ARE mutant enhancer/promoter element derived from the ARE mutant reporter construct was constructed as follows: The ß-lactamase open reading frame from pcDNA6.2-cGeneBlazer (Invitrogen) was isolated by PCR using the following primers:

for 5-GAATCACTCGAGATGGACCCAGAAACGCTGGT-3'

rev 5'-GAATCATCTAGATTACCAATGCTTAATCAGTGAGGCAC-3'

The resulting PCR product was subcloned into pTRED-ARE mut/*luc* (Simmons et al. 2011) between the XhoI and XbaI restriction sites, replacing the luciferase open reading frame and resulting in an ARE mutant-driven ß-lactamase reporter that was confirmed by fluorescent DNA capillary sequencing. A lentiviral vector for ARE-*bla*-mut was generated and titered as previously described (Simmons et al. 2011). HepG2 cells were transduced with ARE-*bla*-mut lentiviral vector at a multiplicity of infection of 10. Cells were allowed to grow in culture for seven days post-transduction to amplify cell number. All cells were cultured in Dulbecco's modified Eagle's medium (DMEM) supplemented with 10% dialyzed fetal bovine serum, 0.1 mM non-essential amino acids, 1 mM sodium pyruvate, and 50 U/mL penicillin and 50 µg/mL streptomycin at 37°C in a humidified 5% CO_2 incubator. All tissue culture reagents were purchased from Invitrogen.

Control well layout for the primary screening

Control wells were arrayed as follows: arrayed as follows: Column 1, concentration response titration of ß-napthoflavone from 46 µM to 1.4 nM; Column 2, 23 µM ß-napthoflavone; Column 3, DMSO only; Column 4, 46 µM ß-napthoflavone. Columns 1 and 3 were identical between the ARE-*bla* and ARE-*luc* primary screening,

while columns 2 and 4 contained 12 µM ß-napthoflavone and 6 µM ß-napthoflavone, respectively, in the ARE-*luc* assay.

Chemical Analysis for Compound Purity

Analytical analysis of the compounds was performed on a Waters Acquity LC/MS (Waters Corporation, Milford, MA, USA). A 2.2 minute gradient of 5 to 100% acetonitrile (containing 0.025% trifluoroacetic acid) in water (containing 0.05% trifluoroacetic acid) was used at a flow rate of 0.5 mL/min. A Phenomenex Luna C18, 2.0 x 100 mm, column with a 2.5 µm particle size was used at a temperature of 45°C. Purity determination was performed using an Evaporative Light Scattering Detector and a Photo Diode Array Detector. Mass Determination was performed using a Waters Micromass ZQ mass spectrometer with electrospray. Data was analyzed using the Waters OpenLynx software. Samples failed to pass QC due to impurities present in the sample or the inability to confirm the molecular weight of the compound with the available resources.

Supplemental Material, Table S1: Activities and potencies for compounds from primary qHTS screen

EC_{50} (µM)	**ARE-*bla* Curve Classification**				
	1.1	1.2	2.1	2.2	Total (%)
<1	3	1	0	0	4 (0.3)
>1 to 10	24	6	6	5	41 (3.1)
>10 to 100	18	7	209	109	343 (25.6)
Total per classification	45	14	215	114	388 (28.9)
% Library*	3.3	1.0	16.0	8.5	28.9

*Based on 1,340 unique compounds

EC_{50} (concentration of half the maximal activity) and efficacy (activation as % positive control) were calculated from the concentration response curves of each individual compound. The four major curve classes (1-4) were defined by previously published criteria (Inglese et al., 2006; Huang et al., 2011; Xia et al., 2008). Briefly, curve classes 1.1, 1.2, 2.1, and 2.2 provide the highest confidence data (and are associated with active compounds), while all non-curve class 4 curves provide lower confidence data (and are associated with inconclusively active compounds). Curve class 4 compounds do not show any concentration response data and are deemed inactive.

Supplemental Material, Table S2: Activities and potencies for compounds from primary qHTS screen

EC_{50} (μM)	**ARE-*luc* Curve Classification**				
	1.1	1.2	2.1	2.2	Total (%)
<1	0	0	2	0	2 (0.2)
>1 to 10	2	1	1	0	4 (0.3)
>10 to 100	1	2	19	16	38 (2.8)
Total per classification	3	3	22	16	44 (3.3)
% Library*	0.2	0.2	1.6	1.2	3.3

*Based on 1,340 unique compounds

EC_{50} (concentration of half the maximal activity) and efficacy (activation as % positive control) were calculated from the concentration response curves of each individual compound. The four major curve classes (1-4) were defined by previously published criteria (Inglese et al., 2006; Huang et al., 2011; Xia et al., 2008) . Briefly, curve classes 1.1, 1.2, 2.1, and 2.2 provide the highest confidence data (and are associated with active compounds), while all non-curve class 4 curves provide lower confidence data (and are associated with inconclusively active compounds). Curve class 4 compounds do not show any concentration response data and are deemed inactive.

Supplemental Material, Table S3: Activities and potencies for 34 compounds overlapping ARE-*bla* and ARE-*luc* assays in the primary qHTS screen

EC_{50} (μM)	**ARE-*bla* (ARE-*luc* Curve Classification)***				
	1.1	1.2	2.1	2.2	Total
<1	0 (0)	0 (0)	0 (0)	0 (0)	0 (0)
>1 to 10	7 (2)	0 (1)	1 (1)	0 (0)	8 (4)
>10 to 100	5 (1)	0 (2)	19 (17)	2 (10)	26 (30)
Total per classification	12 (3)	0 (3)	20 (18)	2 (10)	34 (34)
% Library**	0.9 (0.2)	0 (0.2)	1.5 (1.3)	0.1 (0.7)	2.5 (2.5)

*Numbers in parentheses denote those associated with ARE-*luc* assay
**Based on 1,340 unique compounds
EC_{50} (concentration of half the maximal activity) and efficacy (activation as % positive control) were calculated from the concentration response curves of each individual compound. The four major curve classes (1-4) were defined by previously published criteria (Inglese et al., 2006; Huang et al., 2011; Xia et al., 2008). Briefly, curve classes 1.1, 1.2, 2.1, and 2.2 provide the highest confidence data (and are associated with active compounds), while all non-curve class 4 curves provide lower confidence data (and are associated with inconclusively active compounds). Curve class 4 compounds do not show any concentration response data and are deemed inactive.

Supplemental Material, Table S4: Potencies (μM) and efficacies (%) of compounds from ARE confirmation studies

Compound	Structure	Cluster	ARE-*bla*<br>EC_{50}, μM<br>(Efficacy, %)	ARE-*bla* mutant<br>IC_{50}, μM<br>(Efficacy, %)	ARE-*luc*<br>EC_{50}, μM<br>(Efficacy, %)
1,10-Phenanthroline monohydrate	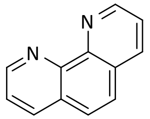	1	inactive	inactive	inactive
1,3-Dinitronapthalene	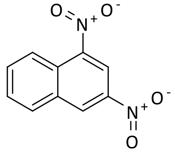	2	1.2 ± 0.2<br>(80)	inactive	16.2 ± 11<br>(180)
2,3,4,5-Tetrachloronitrobenzene	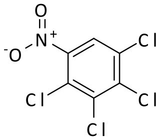	3	9.4 ± 3.2<br>(56)	inactive	27.7 ± 6.7<br>(124)
2-Amino-4-chlorophenol	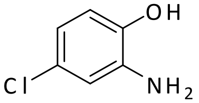	4	7.0 ± 1.8<br>(72)	inactive	6.3 ± 2.1<br>(175)
2-Amino-4-methylbenzothiazole	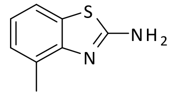	5	10.1 ± 2.1<br>(49)	inactive	inactive
2-Amino-4-methylphenol	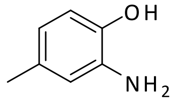	6	12.0 ± 4.8<br>(76)	inactive	24.0 ± 7.1<br>(90)
2-Amino-6-nitrobenzothiazole	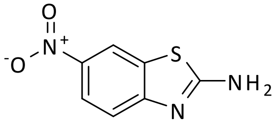	5	16.7 ± 9.6<br>(107)	inactive	inactive
2-Aminobenzothiazole	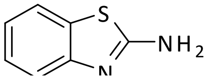	5	inactive	inactive	inactive

2-Chloro-p-phenylenediamine SO_4	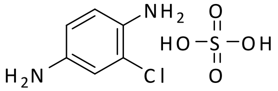	4	17.1 ± 4.1 (72)	inactive	26.5 ± 6.0 (83)
3,5-Dichloroaniline	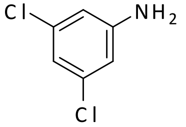	4	inactive	inactive	inactive
3-Dimethylaminophenol	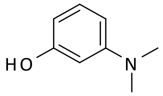	7	inactive	inactive	inactive
4-Chloro-o-phenylenediamine	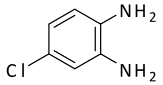	4	inactive	inactive	27.2 ± 3.7 (111.2)
8-Hydroxyquinoline	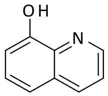	1	16.1 ± 5.1 (53)	inactive	10.1 ± 3.9 (200)
Acetochlor	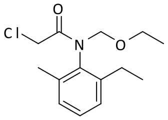	8	4.7 ± 1.6 (86)	inactive	21.9 ± 5.3 (78)
Alachlor	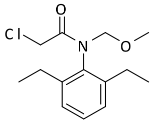	8	5.9 ± 1.7 (106)	inactive	19.9 ± 7.1 (127)
Benzo(b)fluoranthene	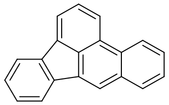	9	5.9 ± 3.0 (136)	23.4 ± 1.6 (134)	1.3 ± 1.0 (19)
Benzo(k)fluoranthene	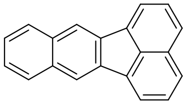	9	1.6 ± 0.7 (114)	18.4 ± 5.1 (160)	4.9 ± 2.2 (103)
Bisphenol A	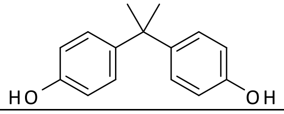	10	12.5 ± 5.6 (48)	inactive	inactive

Cadmium II chloride	$Cl^- Cd^{2+} Cl^-$	11	inactive	inactive	1.1 ± 0.1 (446)
Chlorambucil	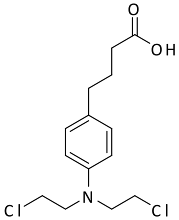	8	inactive	inactive	inactive
Chlorendic acid	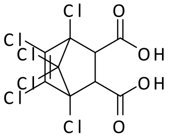	12	inactive	inactive	inactive
Curcumin	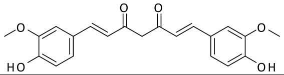	13	3.2 ± 3.2 (21)	inactive	10.8 ± 0.7 (165)
D & C Yellow II	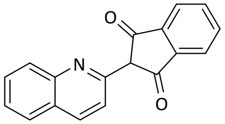	14	0.7 ± 0.1 (88)	3.2 ± 0.4 (48)	1.2 ± 0.7 (185)
Dazomet	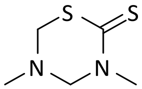	15	20.1 ± 6.6 (55)	inactive	26.6 ± 2.2 (22)
Dieldrin	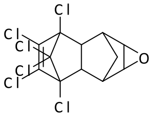	12	15.5 ± 11.8 (40)	inactive	14.2 ± 15.0 (27)
Flavone	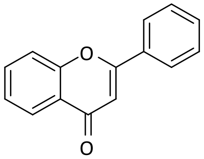	16	2.4 ± 0.6 (80)	inactive	7.6 ± 0.5 (47)
Fluoranthene	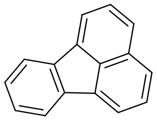	9	inactive	inactive	inactive

Guggulsterones E	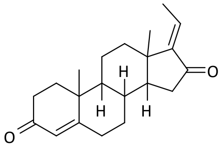	17	5.0 ± 3.1 (106)	inactive	15.4 ± 2.6 (115)
Indium trichloride	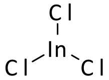	11	14.8 ± 4.9 (92)	inactive	25.0 ± 0 (312)
Iodochlorohydroxyquinoline	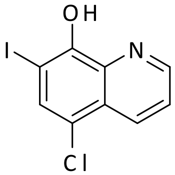	1	17.2 ± 6.0 (41)	19.2 ± 4.6 (21)	10.0 ± 1.2 (147)
Lithocholic acid	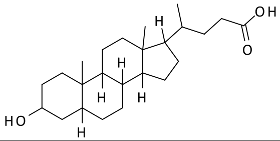	18	17.7 ± 5.7 (65)	inactive	21.9 ± 8.2 (61)
Melphalan	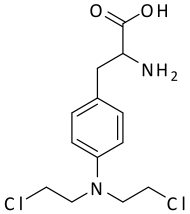	8	inactive	inactive	inactive
n-(1,3-Dimethylbutyl)-n`-phenyl-p-phenylenediamine	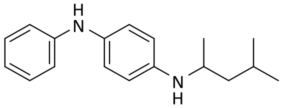	19	17.3 ± 5.0 (89)	inactive	inactive
N,N-Diethyl-p-phenylenediamine	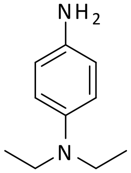	19	18.7 ± 7.6 (60)	inactive	inactive

N,N-Dimethyl-p-nitrosoaniline	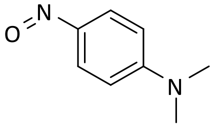	7	9.9 ± 3.4 (82)	inactive	29.2 ± 2.0 (626)
N,N-Dimethyl-p-phenylenediamine	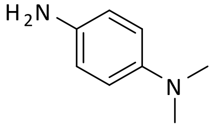	7	9.6 ± 0.6 (80)	inactive	11.8 ± 4.4 (24)
N-Isopropyl-N'-phenyl-p-phenylenediamine	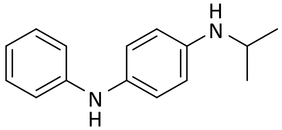	19	13.1 ± 8.9 (71)	inactive	inactive
Nitrogen mustard HCl	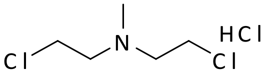	20	10.4 ± 3.6 (46)	inactive	10.4 ± 0.7 (789)
N-Methyl-p-aminophenol sulfate*	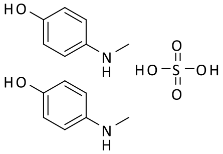	7	13.6 ± 3.2 (37)	inactive	24.6 ± 9.2 (202)
o-Aminophenol	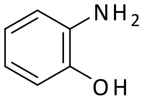	21	6.8 ± 4.1 (95)	inactive	26.2 ± 3.3 (234)
o-Phenylenediamine	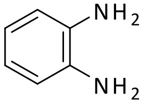	22	7.2 ± 4.1 (72)	inactive	27.3 ± 4.7 (151)
p,p' -DDE	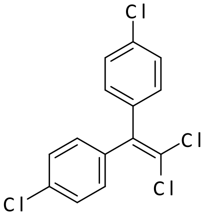	23	inactive	inactive	inactive

Phenylthiourea	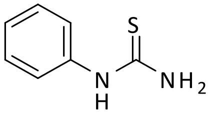	19	inactive	inactive	inactive
p-Nitrosodiphenylamine	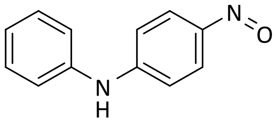	24	9.7 ± 4.1 (31)	inactive	21.7 ± 3.7 (71)
Quinoline	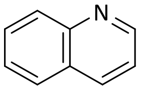	1	inactive	inactive	inactive
Rhothane (TDE)	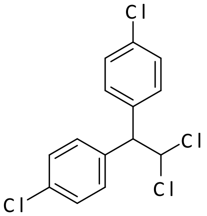	23	16.4 ± 7.3 (43)	inactive	13.5 ± 3.3 (32)
Sodium Cholate	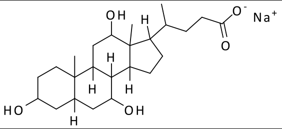	18	inactive	inactive	inactive
Teroxironum	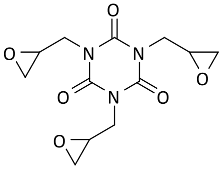	25	5.9 ± 1.9 (119)	inactive	27.1 ± 1.8 (1541)
Tetraethylene glycol diacrylate	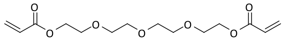	26	9.5 ± 4.8 (53)	inactive	29.2 ± 2.0 (288)
Tetramethylthiuram disulfide	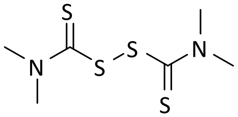	15	17.2 ± 4.9 (35)	inactive	30.6 ± 5.2 (265)

Abbreviations: DDE=Dichlorodiphenyl dichloroethylene; TDE=Tetrachlorodiphenylethane

Each value of potency (EC_{50}, μM) and efficacy (activation of ARE reporter as a % of positive control) from ARE cell-based assays is the mean ± SD of replicates from one (ARE-*luc*) to two (ARE-*bla*) experiments.

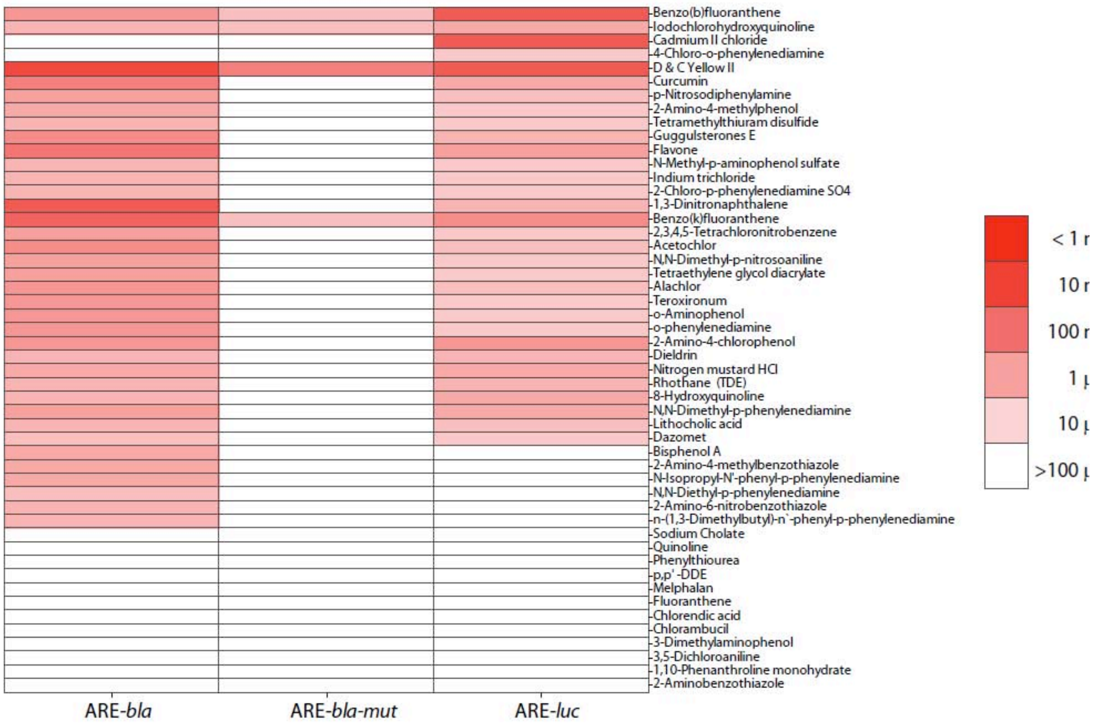

Supplemental Material, Figure S1: Heat map of all follow-up compounds tested in ARE-*bla*, ARE-*bla*-mut, and ARE-*luc* assays. Activity shown was based on log10-transformed compound EC_{50} values across all assays. Each row represents a compound and each column represents a follow-up assay. The heat maps were clustered by pattern and colored based on compound activity, where activity in the assay is colored red, less conclusive activators are colored a lighter shade of red, and inactive compounds are white.

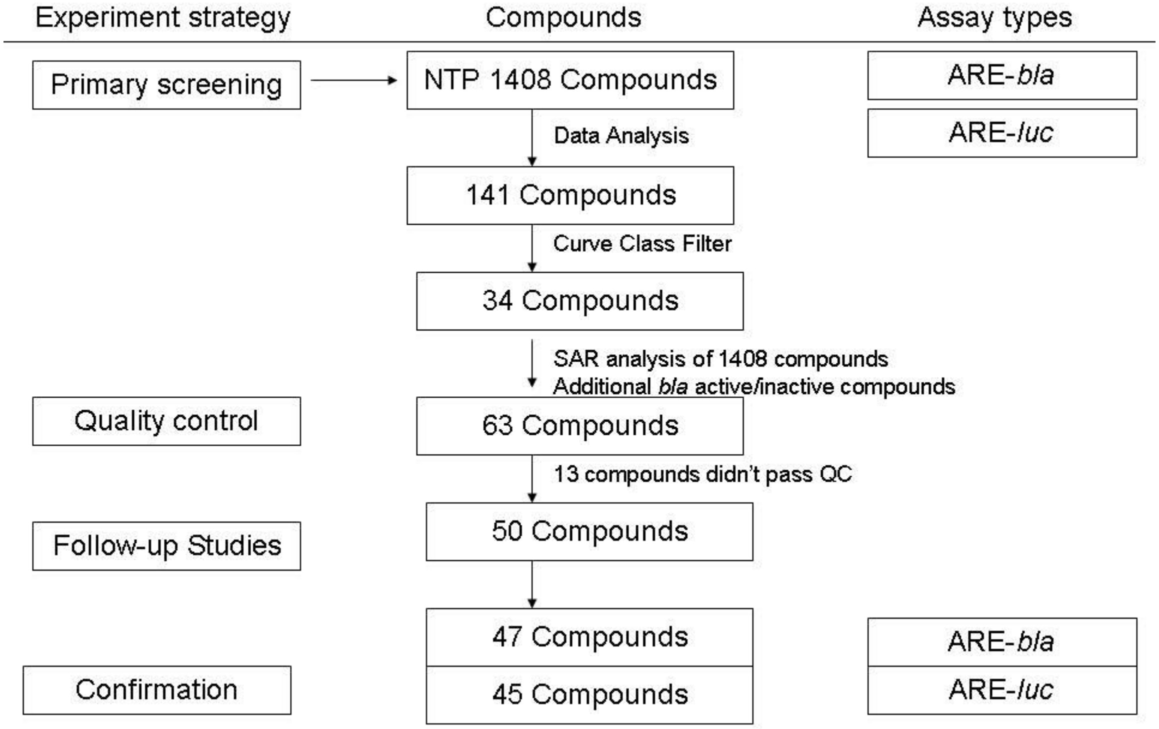

Supplemental Material, Figure S2: A flowchart of identification of ARE inducers in multiple assay formats using qHTS. One hundred and forty-one compounds were commonly active between the ARE-*bla* and ARE-*luc* assays in the primary screen and 34 of those compounds were high quality actives with curve classes 1.1, 2.1, 2.1, and 2.2. These compounds, along with compounds with various activity profiles across different SAR clusters were chosen for follow-up testing, resulting in 63 compounds. Quality control analysis confirmed the identity of 50/63 compounds, hence these were re-tested in the ARE-*bla*, ARE-*luc*, ARE-*bla*-mut and luciferase inhibition (latter 2 assays not shown in the diagram) assays. Forty-seven and 45 compounds confirmed activity in the ARE-*bla* and ARE-*luc* assays, respectively.

References

Inglese J, Auld DS, Jadhav A, Johnson RL, Simeonov A, Yasgar A, et al. 2006. Quantitative high-throughput screening: a titration-based approach that efficiently identifies biological activities in large chemical libraries. Proc Natl Acad Sci USA 103(31):11473–11478.

Huang R, Xia M, Cho MH, Sakamuru S, Shinn P, Houck KA, et al. 2011. Chemical genomics profiling of environmental chemical modulation of human nuclear receptors. Environmental health perspectives 119(8):1142-1148.

Simmons SO, Fan CY, Yeoman K, Wakefield J, Ramabhadran R. 2011. NRF2 Oxidative Stress Induced by Heavy Metals is Cell Type Dependent. Current chemical genomics 5:1-12.

Xia M, Huang R, Witt KL, Southall N, Fostel J, Cho MH, et al. 2008. Compound cytotoxicity profiling using quantitative high-throughput screening. Environmental health perspectives 116(3):284-291.
